# Development of the Community Midwifery Education initiative and its influence on women’s health and empowerment in Afghanistan: a case study

**DOI:** 10.1186/1472-6874-14-111

**Published:** 2014-09-15

**Authors:** Elizabeth M Speakman, Ahmad Shafi, Egbert Sondorp, Nooria Atta, Natasha Howard

**Affiliations:** 1 Faculty of Public Health and Policy, London School of Hygiene and Tropical Medicine (LSHTM), Tavistock Place, London, UK; 2 Rumi Consultancy, Kabul, Afghanistan; 3 Royal Tropical Institute (KIT), Amsterdam, The Netherlands; 4 Kabul Medical University, Kabul, Afghanistan

**Keywords:** Policy analysis, Midwifery, Maternal health, Afghanistan

## Abstract

**Background:**

Political transition in Afghanistan enabled reconstruction of the destroyed health system. Maternal health was prioritised due to political will and historically high mortality. However, severe shortages of skilled birth attendants - particularly in rural areas - hampered safe motherhood initiatives. The Community Midwifery Education (CME) programme began training rural midwives in 2002, scaling-up nationally in 2005.

**Methods:**

This case study analyses CME development and implementation to help determine successes and challenges. Data were collected through documentary review and key informant interviews. Content analysis was informed by Walt and Gilson’s policy triangle framework.

**Results:**

The CME programme has contributed to consistently positive indicators, including up to a 1273/100,000 reduction in maternal mortality ratios, up to a 28% increase in skilled deliveries, and a six-fold increase in qualified midwives since 2002. Begun as a small pilot, CME has gained support of international donors, the Afghan government, and civil society.

**Conclusion:**

CME is considered by stakeholders to be a positive model for promoting women’s education, employment, and health. However, its future is threatened by insecurity, corruption, lack of regulation, and funding uncertainties. Strategic planning and resource mobilisation are required for it to achieve its potential of transforming maternal healthcare in Afghanistan.

## Background

In late 2001, Afghanistan emerged from decades of war with ruined infrastructure and a non-functioning health system [1]. Transition brought national commitment to improving Afghan lives. Crucially, this commitment was supported by the international community with a strong political imperative, technical assistance, and substantial funding [1].

Afghanistan is a traditional patriarchal society, with a restricted role for women embodied in “*purdah*,” a pre-Islamic system prescribing female covering, restricted public movement, and sexual segregation, thus reducing women’s role outside the domestic sphere. During the twentieth century, women’s status gradually changed. By the 1950s, some Afghan women began to achieve university degrees and by the late 1970s, there were around 200 female doctors, engineers and health-workers, particularly in Kabul [2]. However, under the Taliban interpretation of *purdah* (1996–2001), women were denied education, employment, or any public role [2,3]. An under-educated, disenfranchised female population has had serious implications for female healthcare.

Historically, Afghanistan’s health system development efforts were uncoordinated and focussed on major cities, while most of the population was rural. Medical schools were opened in urban centres, but uncontrolled enrolment and insufficient teaching staff meant schools were effectively non-functioning [4]. From 1972, the Intermediate Medical Education Institute in Kabul - renamed The Institute of Health Sciences (IHS) in 2004 - began training health technicians, including midwives [5]. Restrictions on work and education during the Taliban government contributed to no government midwives being trained from 1996 to 2002 [5].

As the century began, after decades of civil conflict, Afghanistan had the poorest health indicators in the region [1]. The Taliban had little administrative capacity, putting religious leaders in charge of healthcare. Health services were underfunded, poorly equipped and staffed, and relied heavily on NGOs. Over 80% of health facilities were NGO-managed when the Taliban government ended in late 2001 [1].

**Figure 1:**
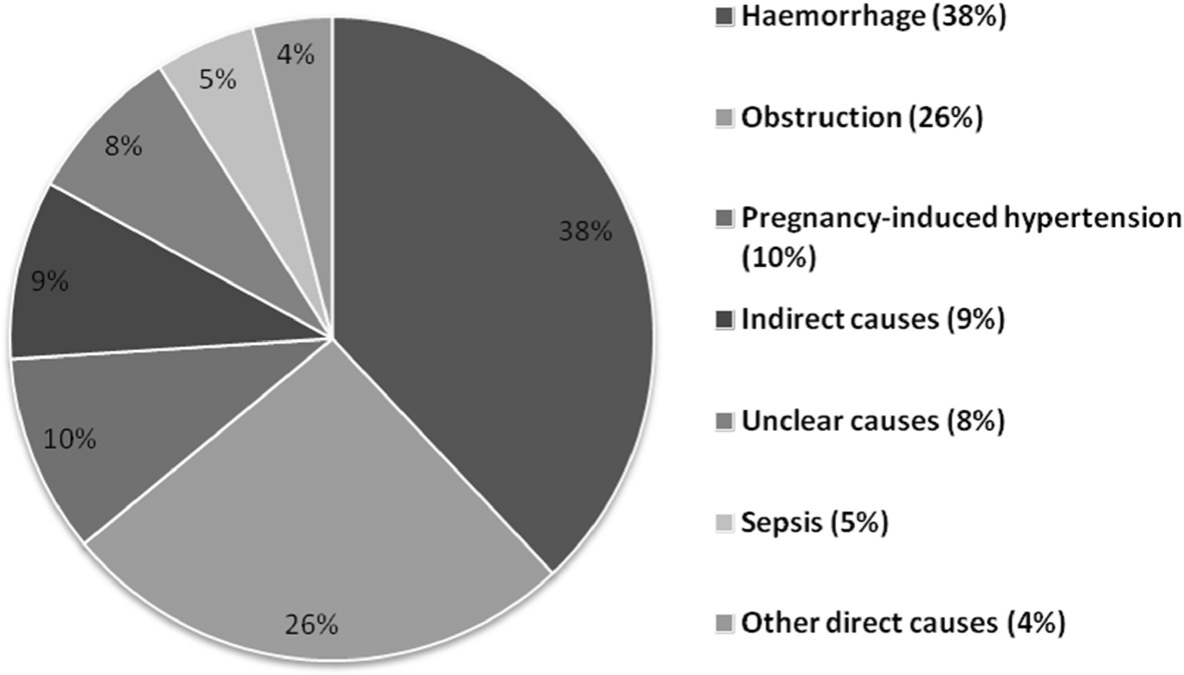
Causes of maternal mortality in Afghanistan (1999–2002).

A key indicator of population health, reflecting the functioning of its health system, is maternal mortality. In 2000, Afghanistan had one of the highest estimated maternal mortality ratios (MMR) in the world of 1,800 deaths per 100,000 live births [6]. An initial assessment in 2002 revealed that Afghanistan had only 467 birth attendants who identified themselves as having at least some formal midwifery training [7] for a population of around 26.8 million [8] and just 8% of women delivered with the assistance of a skilled birth attendant [5,9]. Health facilities remained concentrated in cities, although 75-80% of the population was rural. Figure 1 shows approximately 78% of maternal deaths were from preventable causes, including obstructed labour, haemorrhage, pregnancy-induced hypertension, and sepsis [3].

The impact of maternal mortality goes beyond a woman’s death, frequently leading to higher rates of morbidity and mortality in the immediate family with the primary carer gone. One study found 74% of live newborns subsequently died after their mother died in delivery or postpartum [3]. Most women had no delivery care apart from unskilled traditional birth attendants (TBAs; Table 1) and relied on traditional medicine or ‘*unani tibb’*[10]. Contraception was largely unavailable, leading to an estimated fertility rate of 6.9 births per woman [11].

**Table 1:** Birth attendance terms

**Midwife**	A person who has been regularly admitted to a midwifery educational programme, has successfully completed the prescribed course of studies in midwifery and has acquired the requisite qualifications to be registered and/or legally licensed to practice midwifery (ICM [12]). In Afghanistan, this is a qualification through either the Institute of Health Sciences or the Community Midwifery Education programme.
**Skilled birth attendant**	An accredited health professional (e.g. midwife, nurse, doctor) who has been educated and trained to proficiency in the skills needed to manage normal uncomplicated pregnancies, childbirth and the immediate postnatal period, and in the identification, management and referral of complications in women and newborns (WHO, ICM, FIGO [13]).
**Traditional birth attendant**	A person who assists the mother during childbirth and initially acquired her skills by delivering babies herself or through apprenticeship to other traditional attendants (WHO, ICM, FIGO [13]).

Maternal health experts agree that a key factor in maternal deaths is delivery without assistance from a skilled birth attendant (SBA; Table 1) [14-16]. By 2001, Afghanistan desperately needed skilled female health workers. Particularly, it needed a large number of midwives, trained to international standards and with skills to address the most common birth complications faced by Afghan women. Furthermore, midwives needed to be deployable in rural and often very remote areas where mortality rates were worst. Finally, midwives had to be accepted by communities if they were to change traditional behaviours and improve health outcomes.

Due to these challenges, a new training programme for midwives was initiated and piloted (2002–2004) in eastern Afghanistan and scaled-up nationally in 2005. This initiative became the Community Midwifery Education (CME) programme. Its decentralised implementation made it possible for women to receive training near their own communities and subsequently return to them as trained midwives.

Analysis of policy decisions is important in Afghanistan, as there is intense political and financial pressure to see development initiatives succeed. Afghanistan is the largest recipient of international aid by some margin, and in the context of a global economic downturn, investments must be justified [17]. The aim of this study was to assess how the CME programme developed, implemented, and accepted in Afghanistan. It explores its potential impact on maternal health and more broadly as an example of female empowerment.

## Methods

Policy analysis was informed by a review of published and grey literature triangulated with in-depth interviews with purposively-selected informants. Table 1 provides definitions used for midwife, skilled birth attendant, and traditional birth attendant.

### Data collection

Relevant policy documents, institutional reports, guidelines, and media articles were identified through Google and Google Scholar searches (e.g. using “Afghanistan maternal health”, “Afghanistan health system”, “Afghan midwives”, “Community Midwifery Education Afghanistan” as search terms); hand searches of WHO, USAID, Jhpiego, UNICEF, and HealthNet-TPO websites; and advice from informants. Published literature was identified from PubMed and Embase database searches, using “Afghanistan maternal health”, “Maternal mortality”, “Afghanistan health system”, “Afghan midwives”, “Community Midwifery Education Afghanistan” and combinations of these search terms.

In-depth individual telephone interviews were planned with 5–10 key informants currently or previously affiliated with USAID, WHO, Afghan Midwifery Association (AMA), Jhpiego, or Kabul Maternity Hospital. Potential informants were familiar with CME, either through participating in its development, training or employing CME midwives, or work on maternal health in Afghanistan. Informants were purposively sampled and identified through review of key policy documents and questioning other informants. A topic guide included standard questions, while others were adapted to the status and involvement of each informant. Interviews lasted 1–2 hours, were recorded and transcribed or contemporaneous notes verified with informants. Follow-up email communication clarified statements as necessary.

### Analysis

Policy analysis covers the ten-year period of health reforms by Afghan government and international partners since the end of Taliban Emirate in 2001. Sabatier argued “*‘a decade or more’ is the minimum duration of most policy cycles…*” [18], making this the earliest opportunity to assess CME development and implementation. Qualitative content analysis was used to derive thematic categories from documents and transcripts [19]. Interpretation was supported by Walt and Gilson’s policy framework [20], a descriptive triangle connecting context, content, and process with actors (i.e. stakeholders) central. Authors used these interacting elements, developed specifically for qualitative health policy analysis in low-income settings, to frame CME development and implementation.

### Ethics

All informants received information sheets and consent forms before interviews, with the option to withdraw anytime. Informants were assigned a functional description (e.g. “*international technical adviser*”) for anonymity. The Research Ethics Committee of the London School of Hygiene & Tropical Medicine in the United Kingdom provided ethics approval (reference number 5448).

## Results

Document analysis was supported by primary data from eight key informant interviews (Table 2). Results are framed under sub-headings of context, process, content, and emergent themes (i.e. targets, internalisation, professionalisation, sustainability), with relevant institutional actors discussed under each.

**Table 2:** Key informant characteristics

**Code**	**Title**	**Expertise/Experience**
KI1	International technical advisor	Midwifery expert involved in designing and implementing the CME pilot.
KI2	International public health expert	Scientist who has conducted maternal health research in Afghanistan.
KI3	Senior Afghan midwifery expert	Senior-level midwife, active in initiation of the AMA.
KI4	Kabul-based midwifery consultant	International midwifery consultant at a Kabul hospital
KI5	WHO Medical Officer	Formerly with HNTPO and involved in CME.
KI6	Afghan midwifery trainer	Midwifery expert involved in designing and implementing the CME programme
KI7	Bamyan midwifery trainer	Midwifery trainer in Bamyan province and active in the AMA
KI8	MCH coordinator	With HNTPO during initial discussions about auxiliary midwife training.
KI9	AMA representative (non-respondent)	
KI10	ICM representative (non-respondent)	

### Context

After US and coalition forces ousted the Taliban Emirate, the Bonn Agreement of 22 December 2001 established a six-month Afghan Interim Administration [21]. The Afghan Transitional Administration succeeded it from 13 July 2002 until national elections on 7 December 2004. Transitional leaders were keen to demonstrate rapid improvements and that women had more than a token role in policy-making. As the incoming Health Minister described her appointment [22]:

*"I wouldn't be taking this job if there wasn't a genuine desire for change… I would not have been chosen for this post if women were only being included as some kind of show".* (General Suhaila Seddiqi, Health Minister 2001–2008)

Pressures to succeed included greatly increased international interest, funding, and technical expertise, global health targets, international statebuilding intentions after forced regime change, government desire for legitimacy, and a legacy of conflict, failed governance and gender inequities. These encouraged a supportive environment for piloting women-centred policy initiatives. Political transition and renewed international focus in 2002 contributed to an active and optimistic policy environment conducive to CME development, from groundwork laid during Taliban control.

*“There were so many changes. Change of government, an influx of technical experts, money, and information. The atmosphere was very positive and hopeful and energetic…”* (KI2; International public health expert)

In 2002, due largely to non-functional public infrastructure and grave health needs after decades of conflict and neglect, Afghanistan was an early adopter of government-led healthcare provision through a basic package of health services (BPHS) contracted to non-state providers [23]. BPHS provision, free to the population and prioritising maternal and child health services, was agreed by:

“…*consensus among Afghan Ministry of Health officials, NGOs, international UN agencies, donors and other partners in the health sector.*” (KI2; International public health expert)

While Strong and others argued that the international community initially drove Afghanistan’s health system redevelopment [1], there is no evidence that this went against the aims of MoPH decision-makers. First, data emerging during 2002–2003, showing poorer maternal health indicators than anticipated, encouraged a policy focus on maternal health [3,7,24]. Second, focussing on reducing maternal mortality helped provide legitimacy for the new Afghan government, replacing a government notorious for inequitable treatment of women [2]. Third, global Safe Motherhood policy emphasis on skilled birth attendance and emergency obstetric care (EmOC) provided clear guidance for national maternal mortality reduction priorities [14].

*“It was driven by some very savvy technical assistance… key people who knew what could be done well and quickly and brought that expertise, and it was just readily adopted*”. (KI2; International public health expert)

An influential Lancet-published study, conducted in 2002, estimated a maternal mortality ratio (MMR) for Afghanistan of 1,600-2,200/100,000, ranging from 418/100,000 in Kabul to 6,507/100,000 in remote Ragh district - the highest MMR recorded globally [3]. At the time, maternal health services were minimal, with only 10% of hospitals adequately equipped for caesarean deliveries [1,25]. An MoPH resources assessment found only 18% of all facilities provided delivery services, necessary equipment, and at least one female health worker [7]. In 2002, Amowitz *et al.* estimated an MMR of 593/100,000 in Herat Province [24]. In 2002, there were an estimated 467 birth attendants with some level of training in Afghanistan, ranging from a few weeks to the two or more years required to be certified as a midwife or other skilled birth attendant [7]. Less than 1% of women delivered with SBAs, with most (97%) delivering with TBAs [24].As direct causes of maternal death are largely preventable (Figure 1), policy influencers - including Unicef and WHO - advocated increasing SBA deliveries in Afghanistan to most rapidly increase appropriate interventions and reduce maternal mortality.

*“Midwifery makes a lot of sense, because you can have a midwife out working in 2 years whereas a physician takes a minimum of 6 years. Plus, there’s the extra cost of physicians and the fact that they tend to want to stay more in urban areas…*” (KI2; International public health expert)

Key institutional actors in CME development included transitional government institutions (e.g. Ministry of Public Health, Ministry of Women’s Affairs), international donors (e.g. World Bank, European Commission, USAID), UN agencies (e.g. Unicef, UNFPA, WHO), implementing NGOs (e.g. HealthNet-TPO, Jhpiego), and civil society organisations (e.g. AMA). Dutch INGO HealthNet-TPO (HNTPO) initiated and promoted the idea, developing a pilot programme with technical support from Jhpiego. WHO and the World Bank coordinated the health priorities and funding capacity of the global community, MDG 5 targets of a 75% reduction in maternal mortality from 1990–2015, and mentorship and technical guidance to MoPH.

### Process

Table 3 shows CME groundwork began in the late 1990s, when HNTPO proposed to train ‘*auxiliary midwives’* for rural areas (KI5; KI8). A 1999 HNTPO needs assessment of clinics in Nangarhar province (KI6), and operational qualitative research [26], showed severe shortages of midwives and high reported maternal mortality. HNTPO planned to fill shortages, with Dutch government funding, through an accelerated midwifery-training project targeting rural Nangarhar province (KI5). In mid-2001, the head of the Taliban government granted permission, after lengthy negotiations, and curriculum and selection committees were formed (KI5). However, implementation was delayed by the 11 September 2001 attacks in the US and subsequent regime change in Afghanistan.

**Table 3:** Timeline of community midwifery education, Afghanistan

**1998-2002**	Auxiliary midwife accelerated training program is prepared by Health*Net* International (now HNTPO). Permission is obtained from the Taliban government for a pilot in Nangarhar Province, funded by the Dutch government. The pilot is postponed by forced regime change.
**2002**	The CME proposal is presented to transitional government stakeholders and a pilot programme agreed, funded by the Dutch government with technical assistance from Jhpiego (funded by USAID/REACH).
**Jul 2003**	A workshop presenting first year experience, results in the CME Expansion Programme.
**Aug 2003**	The Guidance Document for Implementation of Community Midwife Education in Afghanistan published.
**May 2004**	Final report published on the pilot project implemented in Ghani Khel, Nangarhar Province (15 June 2002–30 April 2004).
**2004**	The national Community Midwifery Education programme initiated. The National Midwifery Education Accreditation Board (NMEAB) established. Standards and accreditation become mandatory.
**2004-2005**	Formation of the Afghan Midwives Association.
**Sep 2009**	Programme evaluation of the Pre-Service Midwifery Education Programme in Afghanistan.
**Mar 2011**	Evaluation of the Pre-Service Midwifery Education Programme in Afghanistan.
**19 Jan 2012**	Evaluation of midwifery retention in Afghanistan.
**2012**	MOPH Policy updated on Midwifery Education and the Accreditation of Midwifery Education Programmes in Afghanistan.
**2013-2014**	Foundation of the Nursing and Midwifery Council, replacing the NMEAB.

In early 2002, interim policy-makers overwhelmingly approved the CME initiative (KI2). Course length required balancing between accelerated deployment, favoured by INGOs, and ensuring sufficient training for competence, advocated by WHO and MoPH. A 24-month continuous CME training course was approved as a pilot in Ghani Khel, in parallel with (i) expansion of IHS midwifery training to a two-year academic programme through Kabul Institute of Health Sciences and (ii) TBA training. Despite evidence that additional training of TBAs would not reduce maternal mortality, this decision was based on available resources. With very few educated women and approximately 467 birth attendants of varying abilities and certification levels, the cultural norm was that women delivered at home with a TBA or family member and existing human resources nationally were a cadre of unskilled TBAs [27]. TBA training ended after 2005, as planned in the *National Reproductive Health Strategy 2003–2005*[28].

Suitable female teaching staff needed to be identified and in-service training arranged, given the severe shortage of educated women in rural areas. HNTPO staff worked closely with communities and local government. Community leaders were asked to support the search for candidates and advertisements were placed in local media. University of Peshawar staff helped develop the initial midwifery curriculum and Afghan doctors and midwives were subsequently sent there for training-of-trainers. In June 2002, the CME pilot began with Dutch Government funding and Jhpiego technical support to curriculum development.

Student recruitment was from Nangarhar and adjacent provinces according to need (KI3; KI7). Two committees were established to plan student recruitment and deployment, one external and one local. Many of the first students were returnees from refugee camps in Pakistan who had received some education. Students were promised work with HNTPO on graduation and some husbands were also promised work as security guards (KI1). Several informants described the difficulties in recruiting the first group of students, although initial opposition disappeared as communities saw benefits:

“*Sixteen girls were recruited from 4 provinces and came to Nangarhar. It was a struggle to get sixteen and… [we provided] a hostel where they could live with childcare and so on. The families of those 16 were positive deviants…It was also difficult to find girls with enough education – who were literate, could do maths etc*”. (KI1; International technical adviser)

In July 2003, CME was endorsed by MoPH with community midwives recognised as an official cadre. As the success of the pilot became apparent, the programme was scaled-up in rural areas nationwide. ‘*Auxiliary Midwives*’ were changed to ‘*Community Midwives,*’ deemed a more professional title (KI5). New schools were opened by NGOs with technical assistance provided by the Midwifery Education Technical Support Unit (METSU) [29]. In 2005, the Afghan Midwives Association (AMA) was established as a professional association for midwives. Promoting member interests and maternal health strategies, it grew from an initial membership of 15 to nearly 3,000, with chapters in most provinces and an annual national congress.

*So…eventually we got the professional association [AMA] and at the national meeting there were 80 midwives but after that and now, nearly all 34 provinces have a provincial chapter and more than 2,600 midwives. You can’t believe, when you see the national congress of the association, and the wonderful work that midwives are doing, you can’t believe it”*. (KI3; Senior Afghan midwifery expert)

### Content

Midwifery training needed to address the specific maternal health needs of rural Afghanistan. The International Confederation of Midwives model curriculum [30] was adapted during a 2002–2004 pilot by an Afghan midwifery trainer, funded by HNTPO with technical assistance from Jhpiego. A 24-month continuous training course was approved by MoPH and international partners. A competency-based approach focused on development of key skills midwives were required by MoPH to provide:

•the full range of midwifery care, including antenatal, delivery, and postnatal care;

•management of complications, according to basic emergency obstetric and newborn care principles;

•newborn and infant care;

•selected reproductive health care (e.g. contraceptives);

•linkages between women/families/communities/health facilities [31].

A ‘reproductive rights’ approach was adopted, including careful discussion of gender-based violence (KI1). *Islamiat* (Islamic studies) was compulsory, with instruction left to communities. English teaching was included but not assessed. Additional elements were incorporated as the curriculum evolved and training length was reduced to 18 months, after piloting demonstrated that competencies could be achieved within this timeframe.

Students were selected from and by rural communities and agreed to be deployed in their communities on graduating - i.e. based at health centres but providing “*extensive outreach to the community*” [29]. Selection criteria were being female; community approval; aged at least 18 (preferably married with children to gain community respect); ability to read and write and a minimum of class 9 education (although class 6 was accepted for the first 3–5 years); mobility (i.e. able to relocate for 18-month training); motivation; and willingness to adhere to work conditions [29].

Training was conducted in rural health facilities, within a network of basic and comprehensive health centres and a district hospital. Transport and a student hostel with childcare were provided. No more than 20–25 students participated per cohort, allowing an approximate student-teacher ratio of 4:1 [29].

From 2002–2005 the Institute of Health Sciences Kabul (IHS) provided initial accreditation for content and graduate certification. In 2005, the National Midwifery Education Accreditation Board (NMEAB) was established as a semi-autonomous statutory body to guide establishment, performance and accreditation of all midwifery programmes [5]. New CME schools must meet 80% of set national educational standards and be externally reviewed by the NMEAB before they are accredited and able to accept students. At its peak, thirty-two CME schools trained community midwives for all 34 Afghan provinces and, as of 2013, approximately twenty-two were functional [32]. In 2014, the NMEAB is expected to come under the newly created Afghanistan Midwifery and Nurses Council [29].

### Targets and outcomes

Two maternal health indicators used in Afghanistan are MMR and numbers of SBA deliveries. While results for both vary by estimation method, they have improved significantly in the past decade.

**Figure 2:**
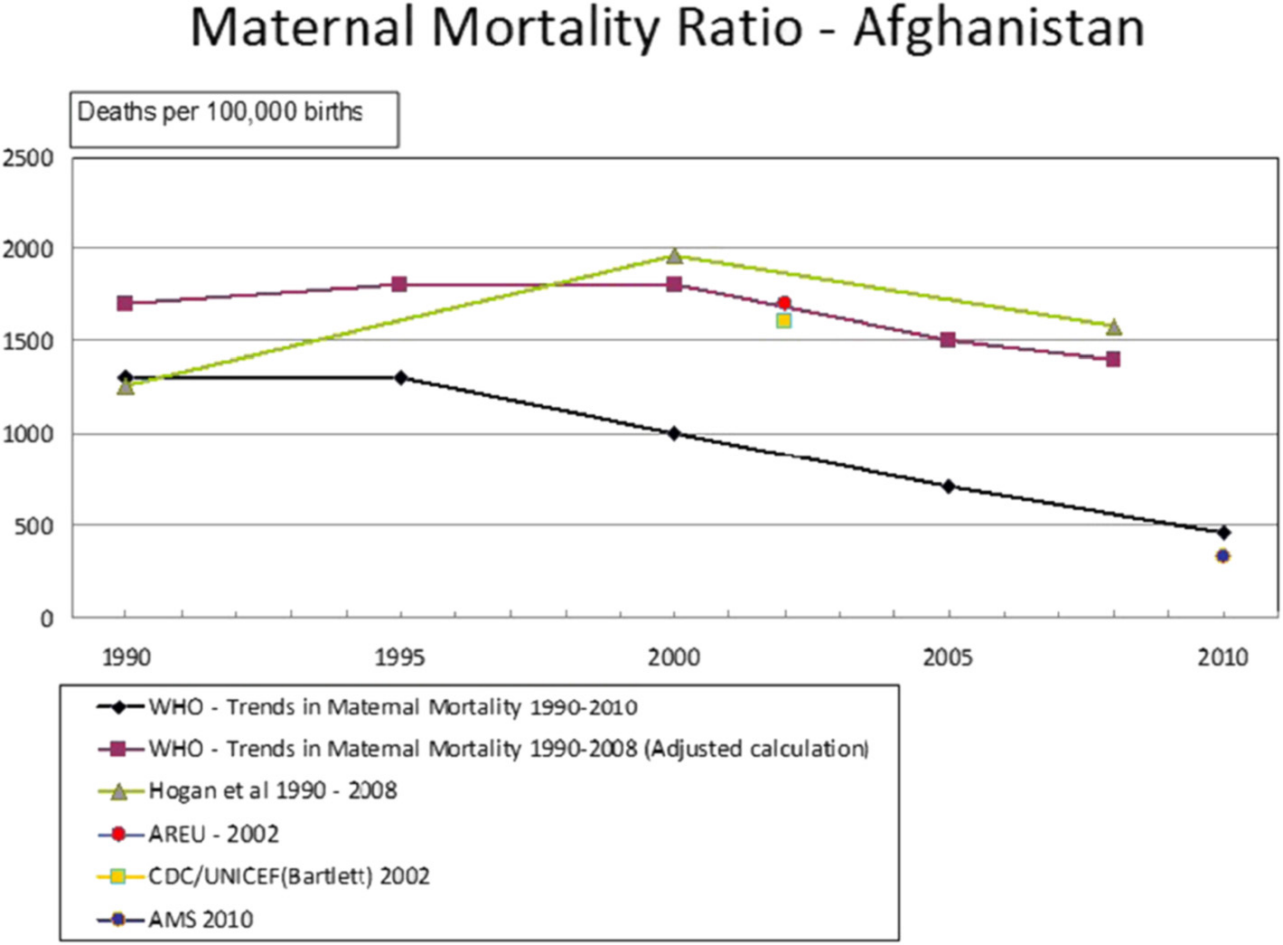
**Reported maternal mortality ratios, Afghanistan (1990–2010).** NB: Adapted from [6,25,27,35,38].

Afghanistan has a revised MDG5 target of a 50% MMR reduction from 1,600/100,000 in 2002 to 800/100,000 in 2015 [33,34]. Figure 2 shows estimated MMR trends using surveys, statistical modelling, and a Reproductive Age Mortality Study (RAMOS). All show downward trends since 2002. The 2010 Afghanistan Mortality Survey (AMS), showing the most dramatic MMR reduction to 327/100,000 and thus successful achievement of the MDG5 target [35], remains contested [36]. Informant views differed. One said any figures accepted by MOH must be correct, while another suggested the AMS figure should be increased by 50-100% (KI2). WHO’s MMR estimate of 1,400/100,000 for 2008 was used in the 2011 State of the World’s Midwifery (SOWM) report, but this was reduced to 400/100,000 in 2013 in the 2014 report [15,37].

Afghanistan has an MDG5 target of a percentage increase in SBA deliveries from 6% in 2002 to 50% in 2015 [34]. The 2010 AMS showed an increase in SBA deliveries to 34% in 2010 [35], while the 2011 SOWM reported 24% [37]. One informant suggested figures remained underestimated, as they excluded home deliveries performed by community midwives (KI7). Government figures suggest the most common reasons for not using SBAs are lack of money and distance from health facilities, as SBA uptake remains highest among wealthier, better-educated, urban women [35]. The AMA argues that some Afghan women will never willingly deliver in health facilities for cultural reasons, proposing to increase coverage through midwifery-led community birthing centres and home deliveries by community midwives. However, some informants favoured the current policy of encouraging health centre deliveries to enable easier referral of complications.

By the end of 2011, the number of new midwives trained under either CME or IHS initiatives was 4,369 recruited (including in training), 3,268 graduated, and 2,721 deployed [39]. More than half (1,470) of deployed midwives were CME trained. Deployment rates are consistently higher for CME than IHS midwives, despite persisting difficulties in insecure areas [32,40]. MoPH committed to increase midwife numbers to 4,556 [41]. USAID estimated 8,000 midwives were needed, a target achievable by 2017–2018 [42]. The 2011 SOWM report estimated 6 midwives per 1,000 annual births would require 9,573 Afghan midwives by 2015 [37]. While one informant said training numbers were on track in 2012, others said targets would not be reached due to reduced donor funding already closing several midwifery schools.

There are 3,268 qualified midwives and a further 300–400 trained annually. If this rate is maintained, excluding attrition it will still take 11–15 years to meet the USAID target of 8,000 and 18–24 years for the SOWM target of 9,573. Simply opening more midwifery schools is insufficient, as coverage must be balanced with quality. However, despite varying MMR, SBA, and midwifery needs estimates, all informants reported the increase in numbers of midwives as a key contributor to maternal mortality reduction.

“*It has clearly been successful. There is a big decrease in maternal mortality, more use of health centres…Midwives are accepted in the community*”. (KI6; Afghan midwifery trainer)

### Internalisation

*Internalisation* is used here to denote the process of acceptance, approval, and ownership among stakeholders as government and civil society take on programme implementation and direction and international guidance reduces. All informants confirmed overwhelming acceptance and responsibility for the CME programme. The lack of entrenched public health and education approaches, due partly to the destruction and chaos of decades of conflict, facilitated rapid acceptance of global best practices among national stakeholders [5]. Ministers, donors, and NGO implementers cite the programme as a success. Government and health donor internalisation was apparent from the expansion from one to 31 midwifery schools, covering all 34 provinces (KI1).

“*Acceptance…was really from across the board from women in communities to the Ministry of Health to donors*”. (KI2; International public health expert)

All informants praised the CME model as an effective means of training midwives for deployment in rural Afghanistan. Frontline implementers described community acceptance and ownership.

*“From the very beginning this programme was very well accepted by the community…if we needed 22–24 students for a batch, we got 100 applications and later on 300 applications… All people wanted to send their daughters, their sisters. And the community… realised that they needed female health workers for reproductive health services…and later on when the midwives contributed to the family’s income it was another value added”.* (KI3; Senior Afghan midwifery expert)

Empowerment was a frequent theme among informants. Some highlighted the social effects of educated midwives providing a positive role model for women and girls.

“*Many of the communities had never had an educated woman living there. And now they have an educated woman who helps these women at the most important time of their lives, giving birth. And one of the quotes…was that ‘we’ve seen these midwives and it motivates us to send our daughters to school’. So the impact is broader than just direct healthcare provision*…” (KI2; International public health expert)

Several described increased female empowerment in relation to SBA usage.

*They got a voice, they got power…In one health facility, because of a midwife the numbers of deliveries in the health facility had increased from 2–3 per month to 50–60 per month…Women didn’t go before because there wasn’t a female provider or a qualified midwife for them…it’s having a midwife who makes a partnership with women and this makes a difference…*” (KI3; Senior Afghan midwifery expert)

Others described CME trainer and student empowerment.

“*Local Afghan midwifery leaders have come up and have really taken to looking up research online and going for training abroad and bringing that knowledge back to the midwifery community for continuing education here…So in the last five years things have progressed quite a lot*”. (KI4; Kabul-based midwifery consultant)

### Professionalisation

Professionalisation remains a challenge. Afghan midwives are either Community Midwives, trained in provincial schools to work in rural areas, or IHS midwives, trained and usually deployed in cities. Programme differences led to misconceptions among both policy implementers and service users:

•Entry requirements - because of the desperate shortage of educated girls in rural areas, entry requirements were reduced to Class 6, later raised to Class 9. Entry to the IHS programme required at least Class 9, later raised to Class 12;

•Training length - IHS training lasted two academic years, while CME was initially eighteen months continuous “*to accelerate deployment to needy communities*” [43].

Informants were clear that lower CME entry requirement were a pragmatic response to insufficient human resources, rather than reflecting an easier programme. Course time was initially equivalent. An additional six months, added to the CME curriculum in 2010, made it longer and more comprehensive than IHS training [44]. CME midwives consistently outperform IHS midwives in competency tests [45], attributed to greater motivation, more practical experience, and better quality teaching. However, several informants spoke of doctors failing to acknowledge the abilities of qualified midwives and discouraging them from performing tasks for which they were capable. A perception among provincial authorities remains that IHS midwives are more qualified and competent than CME midwives (KI1; KI3). Most informants suggested that merging the IHS and CME programmes would finally eradicate this discrimination.

Informants confirmed that status, recognition and a clear career path are important to community midwives. Some have already left the profession because of dissatisfaction (e.g. CME midwives being considered less qualified than IHS midwives, not being permitted to fully use their skills, those without secondary school qualifications not being accepted as civil servants).

The AMA has become the mouthpiece for midwives. The AMA was founded to professionalise midwifery and help address disparities and has grown exponentially. Community midwives are a new cadre and AMA initiatives include:

•a “bridging programme” to higher education, negotiated with the Ministry of Education, to resolve the career block for CME midwives unable to complete secondary school and thus excluded from the civil service;

•midwife-run community birthing centres;

•CME professionalisation with defined career progression.

All informants spoke of the dynamism and commitment of the AMA, a positive sign of programme internalisation and hope that a critical mass of educated women will keep the profession strong.

*“At the initial meeting on 29 July 2004, there were 15 midwives and I asked them why we needed this association. And everyone was talking: ‘we need to be respected, we need professional development, we need rights, we need to be recognised as a midwife and midwifery needs to be recognised as a distinct profession’. So, it was mainly for a personal career path in professional development and to be autonomous”.* (KI3; Senior Afghan midwifery expert)

### Sustainability

Sustainability issues included security, corruption, quality-maintenance, and perhaps most importantly financing. Opinions on security were mixed. Although one midwifery school deploys midwives to Taliban-controlled southern provinces, many midwives are unwilling to work there because of personal risks, family opposition, and potential to be the only female health-worker in their catchment. While one informant called political insecurity the biggest obstacle to progress, most noted security had not prevented CME midwives working in all provinces. However, all said the situation was worsening as the Taliban regained strength.

Opinions were mixed regarding the effects of corruption (e.g. unfair programme acceptance, altered exam results). Some argued it was a minor problem, limited to some nepotism, while others said it threatened to undermine the whole programme. International commentators have criticised the donor programme for heavy wastage, due to mismanagement and corruption within the Afghan government [46]. The Afghan government has said money is being squandered on International NGOs, high salaries to Western advisers, with over 80% never reaching Afghan recipients [47]. Informants agreed high-level wastage resulted in money lost to smaller development programmes including CME.

Afghanistan’s regulatory system remains weak with a significant risk of midwifery schools opening without proper capacity or quality-assurance (KI1). An Afghan precedent was the 1990s proliferation of substandard medical schools [4]. Afghanistan has no history of professional standards and corruption appears to be increasing (KI1). As lack of regulation could undermine the future of the programme, MoPH has approved an Act to establish a new regulatory body - the Nursing and Midwifery Council, expected to be in place by 2014. Its powers and expected effectiveness remain unclear.

Informants reported programme financing as crucial and increasingly challenging, with donors reducing funding and some schools already closing. It costs US$9000-10,000 annually per CME midwife trained with additional funding needed for deployment [40,48]. Afghanistan’s health system remains 90% dependent on foreign funding [49]. Afghanistan’s economy has been growing steadily, but still lacks resources and administrative capacity to manage without aid [50].

“*There’s no discussion at the moment about the transition to the MoPH and sustainable health financing…We know that the MoPH currently do not have the capacity to provide [healthcare], despite system strengthening and a lot of capacity building, they just cannot deliver healthcare*”. (KI1; International technical adviser)

However, the focus of aid has changed since 2002, with increasing gaps in provision [51].

“*What is going on now is that donors have stopped the funding so midwifery programmes close down…When the fund is finished, they don’t have money, even if there’s a strong need…We never closed a midwifery education programme because of security, but the programme closed because of lack of funds…*” (KI3; Senior Afghan midwifery expert)

## Discussion

Maternal health indicators have improved since 2002 (Figure 2). However, even choosing the most favourable statistics, MMR remains high at 327 and SBA deliveries low at 34%. With a 2012 fertility rate of 6.3 and contraception used by 19% of reproductive age women [37,52], increased family planning coverage is an important means of improving maternal health to which community midwives could contribute [53].

Ascertaining maternal mortality is difficult without civil registration of births and deaths, large areas of the country inaccessible, shortages of medical personnel to verify causes of death, an under-educated population, sensitivities associated with maternal death, and political pressures to present favourable results. Despite unreliable health indicators, evidence shows the CME programme and community midwives are widely accepted as an integral part of the Afghan health system by government, international partners, and communities. CME has empowered women by:

•educating them to play a crucial role in society and the workforce;

•employing women, who gain economic independence and contribute to family incomes;

•encouraging female education by showing it can lead to economic opportunities;

•prioritising women’s health needs in communities.

As the voice of Afghanistan’s midwives, the AMA can strengthen empowerment initiatives - particularly if given a more public role (e.g. in the National Health Strategy, in health sector training). AMA initiatives provide a model for ambitious Afghan women in education, business and other sectors, while long-term follow-up of CME graduates can help the AMA ascertain deployment, career paths, and refresher training needs.

Security issues continue to challenge deployment, demand, and data collection. Lower deployment in insecure provinces means inequitable maternal healthcare provision, weakened data reliability, and potentially underestimated mortality. MoPH could make deployment safer and more attractive (e.g. through enhanced salaries, a higher ratio of midwives to population in insecure areas, negotiated protection with local leaders).

While coverage is still the primary objective, as midwife numbers increase greater emphasis should be placed on quality. While strong regulation is needed to support training quality, most informants considered it premature to transfer responsibility entirely to Afghan bodies, suggesting sharing it between national and international experts. A gradual reduction in midwifery school numbers could help ensure a high standard of teaching and potentially lead to the ICM’s aim of midwifery degree courses [3]. There is pressure on the Afghan government to take over health system funding and MoPH is encouraging private sector involvement [54]. Although midwifery services through the BPHS are designed to be free for users, there are indications that fees are still charged in some areas [23,54]. Service-related midwifery costs could increase inequities. Healthcare usage is already highest among wealthier urban women and travel and other costs have been cited as a reason not to use health facilities [23,32,35].

Document review and interviews were limited by language, access, and time. Several purposively selected informants were key actors in CME development and strongly supportive of the initiative. A larger sample could have provided greater diversity of perspectives. However, interviews were intended for data triangulation and comments were not found to differ from documentary evidence.

Lessons from the CME programme may be applicable elsewhere, though care should be taken to adapt these to local contexts [40].

## Conclusions

CME began as an NGO pilot initiative and developed into an internationally-recognised programme. The programme is considered a success by stakeholders, with one informant attributing this to “creative, energetic people from the beginning”. Many factors contributed, including NGOs on the ground, readily accessible funding and international technical expertise, regime change enabling early policy debate, establishment of regulatory and accreditation bodies, and crucially, community midwives ready to become community role models.

CME is already being used as a model for other countries (e.g. Pakistan, Ethiopia, Laos). However, the initiative is only a decade old and still potentially fragile. It has benefitted from intensive financial and technical inputs unsustainable in the long term. It was developed in a challenging environment including cultural norms that may take generations to change. There are major threats, primarily security and financing - with related risks of corruption and weak regulation affecting quality. Sustainability depends on the Afghan government internalising the programme (e.g. by including CME-trained midwives in the civil service).

CME has surpassed its original goal, is concerned with both female healthcare and female education, and has had a wider social influence than was anticipated. MDG5 may not be reached in 2015, as improvements are inconsistent throughout the country. However, it is clear that there are improvements. MMR reductions are likely due to a range of initiatives, including CME. CME has improved maternal care and provides an example of female empowerment. Midwives are respected members of the working population. It is vital that this role is nurtured, no less than their role in reducing the preventable deaths of so many Afghan women every year. This is clearly a long-term process.

## Competing interests

The authors declare that they have no competing interests.

## Authors’ contributions

EMS conceived and designed the study, acquired and analysed data, and prepared the manuscript. AS and ES contributed to data acquisition and interpretation and critically reviewed the manuscript. NA contributed to data acquisition and critically reviewed the manuscript. NH contributed to study conception and design, data analysis and interpretation, and critically revised the manuscript. All approved the final version and agreed to be accountable for the work.

## Pre-publication history

The pre-publication history for this paper can be accessed here:

http://www.biomedcentral.com/1472-6874/14/111/prepub
